# Whose Copy? Whose Rights?

**DOI:** 10.1371/journal.pbio.0020228

**Published:** 2004-07-13

**Authors:** Andy Gass, Helen Doyle, Rebecca Kennison

## Abstract

Primary scientific and medical literature is written for academic communities and the public good -- so should be governed by copyright licenses that permit the full range of uses that could benefit stakeholders in research.

*This is the third installment in a series of editorials on the implications of open-access publishing for established publishing practices and stakeholders in scientific and medical research.*

The questions, tensions, and social concerns surrounding copyright and the Internet are very different for scientific and medical literature than for other kinds of easily reproducible digital works. Peer-reviewed publications are often the sole tangible products of the tremendously time-consuming and expensive process of conducting primary research in biology and medicine. Who should own the primary research articles that are the culmination of years of work by scientists and staggering financial investments by governments, universities, and tax-exempt foundations? What uses should the documents' owners permit?

In practice, academic authors typically assign full copyrights to their articles to the publishers of the journals in which the works appear. Scientists do not benefit financially from the transaction; indeed, they often subsidize the cost of their articles' publication in the form of page charges, color charges, and other fees levied by publishers. The prestige associated with publishing in a selective journal is sufficiently valuable that scientists are generally willing to abdicate the legal rights to their own work without remuneration.

In recent years, however, several technological and legal innovations have led a growing number of scientists to begin to question the sagacity of this arrangement. The advent of electronic publishing and the Internet itself have made technically possible a slew of novel uses of primary research papers. Simultaneously, the traditional “all rights reserved” copyright license has been supplemented by a variety of alternative licenses—of equal legal validity and available at no charge to anyone who wants them—that allow copyright holders to prevent some uses of a work without permission, but to authorize others. Different licenses created by the nonprofit organization Creative Commons (www.creativecommons.org), for example, allow copyright holders to mark their work with freedoms—to permit a work's reproduction for any noncommercial purpose (the Noncommercial License) or for any purpose at all provided that the original authorship is properly attributed (the Attribution License).

The upshot of these developments is that copyright holders can now permit a spectrum of uses of a paper by prospective researchers, anthologizers, archivists, teachers, patients, policy makers, journalists, and other interested parties. Precisely which uses are permitted and which are not is far from a trivial matter. The particular copyright license under which an article is published largely determines how the document can be stored, searched, and built upon by other scientists.

## Authors' Rights and Users' Rights

One implication of the variety of copyright licenses now widely available is that the right to use an article in one way or another is largely independent of its accessibility online. A paper that is touted as “freely available” or “free access” is very different from one that is “open access.” (See http://www.plos.org/openaccess for the formal definition of an open-access article, drafted at the 11 April 2003 Bethesda Meeting on Open Access Publishing.) When a document is “freely available,” someone who comes across it may be permitted to do nothing more than read it online on a publisher's Web site; the right to use the article in any other way is typically granted only at the publisher's discretion. When a document is open access, however, a wide range of additional uses are perpetually and irrevocably allowed— from the reproduction and distribution of the paper by a professor for his or her students to the archiving of the paper in a searchable online repository available to anyone in the world with an Internet connection, and more.

The Creative Commons Attribution License (CCAL), which governs this editorial and all other content in *PLoS* *Biology*, permits a number of uses of articles that are typically restricted and for which there is an immediate demand. All articles published by PLoS can be included in coursepacks—a use-right that most authors would want to allow without exception, but that most traditional publishers grant only for a substantial fee (which they rarely share with authors). The CCAL also ensures that institutions are permitted to archive not only articles written by their own faculty, but all other works published under the same legal terms as well, thereby facilitating their permanent accessibility and preservation. For example, the LOCKSS (“Lots of Copies Keep Stuff Safe”) program (http://lockss.stanford.edu/projectdescbrief.htm), an ongoing project to support libraries' efforts to “create, preserve, and archive local electronic collections,” is viable only insofar as institutions are permitted to store information themselves, rather than access it exclusively via publishers' Web sites. Many collaborative projects between libraries and publishers have been complicated by legal constraints, including the stipulation that archives remain “dark,” or inaccessible to users, until any commercial incentive for restricting access to articles has been exhausted—clearly a suboptimal arrangement for researchers, and one that is unnecessary for collections of works governed by the CCAL.

One of the truly revolutionary implications of open-access articles, however, is that we simply do not know the full range of their potential applications. They are available for any use that any entrepreneur can envision, so long as the authors of the papers are properly credited. The only certainty, then, is that the utility of open-access research articles will be limited solely by the imagination of those that are inspired by the possibilities—rather than by legal constraints.

## Authors' Protections

Authors retain the copyright to all articles in *PLoS Biology* and license their works under nonexclusive terms that reserve only some—rather than all—rights. There are several common objections, generally leveled by publishers, against this practice. For example, it is sometimes argued that the traditional copyright arrangement in scientific publishing protects against uses of articles that authors would object to—while the CCAL permits such uses and renders authors helpless to prevent them.

To the extent that the uses in question are for academic or archival purposes, such as those discussed above, it is certainly true that the CCAL permits practices that “all rights reserved” licenses do not. Indeed, the expanded range of legitimate academic uses of articles is among the primary selling points of the CCAL in the context of scientific publishing. To the extent that the ostensibly objectionable uses are commercial, the problem is easily remedied with the Creative Commons Noncommercial License, which prohibits commercial reuse of a work without the copyright holder's consent.

PLoS has chosen, for reasons both philosophical and pragmatic, to permit the commercial use of works we publish. As a matter of principle, all of our policies reflect the view that scientific publishers are service providers and should not themselves restrict the potential applications of the largely publicly funded work in their journals. More concretely, if a commercial enterprise is interested in repackaging the articles that PLoS has published, we are loath to prevent an author's work from wider distribution. Any risk that a company will use an article for a purpose its author would be uncomfortable with is, in our view, substantially outweighed by the benefits of allowing—not on a case-by-case basis, but across the board—the reproduction of the article for inclusion in online encyclopedias, or for distribution in countries in which Internet access is unreliable, or, indeed, for creative uses we hope to inspire by making primary research articles legally available to commercial interests.

Another recurring objection to the copyright arrangement that PLoS employs is that authors are inappropriate copyright holders because they are ill-equipped to protect their own works against plagiarism, misattribution, and other misuse. Most scientists, however, have enough familiarity with cases of plagiarism in their own field to know that their strongest protection against mis- or nonattribution is derived not from the threat of prosecution for copyright infringement, but from community standards of conduct. Furthermore, among the benefits of open-access articles is the fact that their full texts, rather than just their abstracts, are searchable—which, as any teacher knows, makes plagiarism much easier to detect.

Beyond plagiarism and misattribution, it is not clear what uses of primary research articles authors would actually want to prevent (other than, perhaps, the commercial uses that their work is already susceptible to, in many cases, when publishers hold copyrights). Scientists do not receive royalties for their published work. The more widely their articles are read and cited, the more their professional reputations are bolstered. Certainly, research articles have a wide range of uses that publishers typically object to— and indeed often file suit over—such as their compilation in coursepacks by copy shops. Those applications, however, tend not to constitute “misuse” in many authors' eyes.

## Authors' Voice

There is no question that the licensing arrangement PLoS employs is relatively novel—and therefore untested over the long term—in biomedical publishing. However, it hardly takes a radical understanding of the interests of authors and users of primary research articles to conclude that the open-access terms of copyright promise substantial benefits for both groups.

What, then, can scientists do to encourage other publishers to follow suit and strike similar legal arrangements with authors as a matter of course? One answer is to “vote with your submissions;” that is, authors should submit their work preferentially to journals with copyright and licensing practices that genuinely serve their interests. Another equally important action for scientists is to raise the issue with their professional societies. Scholarly associations exist, among other reasons, to serve the needs of their members—and society members should actively urge their society journals to employ the CCAL or a similar license for their research articles.

Scientific and medical literature is different from fiction or movies or music. The United States government invests more than $28 billion per year in the National Institutes of Health alone to fund research in biology and medicine. The scientists who conduct that research and the research paid for by other public-minded institutions in the United States and abroad have an affirmative moral obligation to share the knowledge they create—not just with students and faculty at elite Western universities, but with everyone who could use it and build upon it. When authors publish their work in journals with restrictive copyright practices, it becomes illegal (often for even the authors themselves) to store primary research articles in many archives or include them in coursepacks or use them for other responsible purposes. Those obstacles to sharing knowledge can be avoided without legislative intervention, however, if scientists and publishers alike embrace a legal paradigm for disseminating new discoveries that maximizes their utility.

